# An Investigation of the Mechanical Properties of Ti Films Reinforced with Wood Composites by Growing Ti Particles on a Wood Substrate

**DOI:** 10.3390/polym17050583

**Published:** 2025-02-22

**Authors:** Wenhui Bao, Yini Tan, Ziyi Ying, Rui Xue, Xiaojiang Xu, Shuangping Duan, Haizhuan Lin, Hui Chen

**Affiliations:** 1 College of Architecture and Energy Engineering, Wenzhou University of Technology, Wenzhou 325035, China; nefuwenhui@163.com (W.B.); linhaizhuan@wzut.edu.cn (H.L.); 2 Wenzhou Key Laboratory of Intelligent Lifeline Protection and Emergency Technology for Resilient City, Wenzhou 325035, China; 3 Key Laboratory of Bio-based Material Science & Technology, Northeast Forestry University, Ministry of Education, Hexing Road 26, Harbin 150040, China

**Keywords:** wood, Ti film, magnetron sputtering, laser vibration measurement, dynamic rebound test, dynamic mechanical analysis

## Abstract

Table tennis racquet blades (TTRBs) are specialized wood materials known for their excellent mechanical properties. As one of the widely used physical vapor deposition technologies, magnetron sputtering has become the most effective method for preparing various thin film materials. In this study, the surface of the TTRB is coated with a Ti film with different thicknesses by magnetron sputtering to improve the performance of the TTRB. The surface roughness, crystal structure, viscoelasticity of the TTRB were analyzed by means of non-contact surface profilometry, X-ray diffraction (XRD), and dynamic mechanical analysis (DMA). In order to effectively test TTRB properties, three types of testing devices were designed, including free-fall rebound, laser vibration measurement, and the dynamic rebound test. The results reveal that the deposition of a Ti film on the surface of the TTRB improves the rigidity and rebound efficiency of the TTRB. Under optimized conditions, the initial amplitude, vertical rebound distance, and rebound rate can reach 2.1 μm, 23.7 cm, 13.7%, respectively, when the deposition thickness is 5 μm. It is anticipated that the modification and the corresponding detection methods developed in this study can foster innovative product development, standardize the TTRB industry, and contribute to the advancement of table tennis.

## 1. Introduction

**Figure 1:**
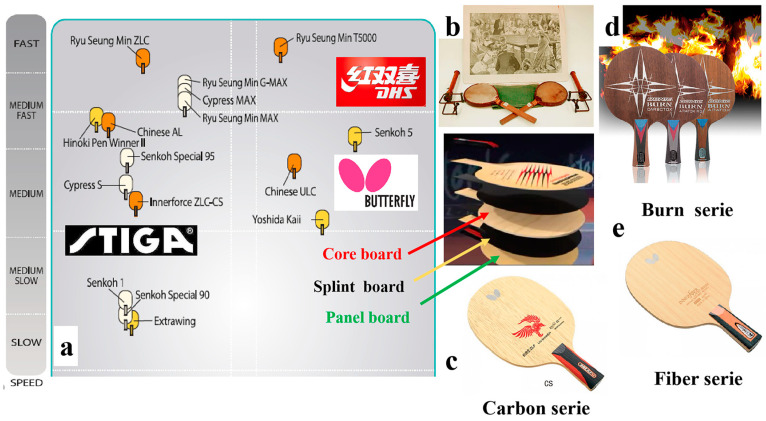
(**a**) The TTRBs are classified according to the subjective feel of the athlete. (**b**) The equipment of the initial origin of table tennis. (**c**) The schematic illustrations of the TTRB by adding a carbon layer. (**d**) The schematic illustrations of the TTRB hardening at a high temperature. (**e**) The schematic illustrations of the TTRB using man-made fibers.

As a renewable biomass material, wood is widely used in daily life and primarily serves engineering purposes [1,2]. Although various new buildings and decorative materials have been developed [3], wood remains irreplaceable in some specific areas of application, such as in music and sport fields, due to its unique mechanical [4,5], physical [6,7], and esthetic properties [8,9]. As one of the well-known Olympic games [10], table tennis has been a globally recognized sport for over 100 years, appearing not only in everyday life but also in international competitions. A table tennis racket is composed of table tennis racket blades (TTRBs), sponge, and rubber [11]. A TTRB commonly consists of a single layer of wood veneer, formed through gluing and compression molding [12,13]. More commonly, it is made from five-layer or seven-layer blades. The purpose of the singular plywood is to create a core layer at the center of the TTRB, referred to as the core board. The thickness of the core board typically ranges from 2.5 to 5 mm. On both sides of the core board, there are the splint board and the panel board, as illustrated in Figure 1a. The splint board, also known as the force material, has a thickness of 0.5 to 1 mm and is used to balance the force conduction between the panel board and the core board. The combination of the harder surface material and the softer force material enhances control performance during play, allowing for the better execution of various batting techniques. A thicker force material results in a faster ball rebound from the blade but reduces the ability to absorb the ball, leading to less control. The panel board is the thinnest layer, with a thickness ranging from 0.2 to 0.5 mm, and the selection and thickness of the materials for each layer play a crucial role in determining the overall softness and hardness of the TTRB. A TTRB is generally classified into two types according to the way of holding, i.e., the horizontal plate and the straight plate. The horizontal plate can be further divided into a straight handle and a waist handle. The straight racket handle is particularly conducive for fast attack techniques such as smashing, dunking, and slicing. In competitions, the waist-shaped handle is commonly used by athletes who specialize in fast-paced attacking play. This is because the front section of the racket’s bottom plate is thinner, while the tail is wider and thicker. This design provides athletes with a unique grip sensation, offering a balance of looseness and tightness, which enhances control and maneuverability during fast attacks. Since the end of the 19th century, the original TTRB was made of a paper material (Figure 1b) and has gradually evolved to incorporate canvas, glass sandpaper, and multi-layer natural wood [14]. Nowadays, with the emergence of new materials, as well as changes in game rules, athletes increasingly prefer lightweight and flexible TTRBs [15]. Manufacturer have improved the properties of TTRBs by adding materials such as a carbon layer (Figure 1c), man-made fibers (Figure 1e), and photosensitive resin to the blades [16]. Overall, the manufacturer’s innovation mainly focuses on three aspects: shape design, material matching, and adhesive development, but the breakthrough progress in these areas is very slow [17].

From the perspective of wood modification [6,18], modified wood is typically defined as any wood that has undergone chemical [19,20], physical [21,22], or thermal processes [23,24] to enhance its properties [25]. The modification process can improve the dimensional stability of wood [26], reduce its hydrophilicity, and affect various mechanical properties (e.g., hardness) [27]. Today, several wood modification techniques have been commercialized, including acetylation [4], furfurylation, charring, and thermal modification [8,28]. The Burn series is a mainstream product produced by DONIC Co., Ltd. (Verklingen, Germany). (Figure 1d). The stiffness and dimensional stability of the TTRB increased after treatment at 200 °C for 24 h [29]. The carbon fiber series is the mainstream product of STIGA Co., Ltd. (Stockholm, Sweden). The mechanical properties of the TTRB are improved by adding artificial fiber cloth to the wood. Butterfly is also one of the manufacturers of high-end table tennis supplies, with its ZLC series featuring high-density woven carbon fiber in the TTRB. In addition, manufacturers also focus on the production process, glue types [30], handle design, material collocation, and other aspects of TTRB innovation. Last year, the DHS Company approached our lab to chemically modify TTRBs, aiming to reducing the cost of production by replacing precious wood with low-quality alternatives. Therefore, the goal is to enhance the performance of low-quality wood TTRBs to meet the demands of high-level athletes. The approach we adopted was to create rigid structures by immersing the wood in a low-molecular-weight resin solution, leveraging the crosslinking of the resin with the hydroxyl groups in the cell wall of the wood. The results showed that the weight of the chemically modified TTRB ranged from 150 to 160 g, while the weight of a commonly used TTRB was between 75 and 90 g. A 150 g table tennis racket base is too heavy for a professional athlete. Although the elastic modulus of the chemically modified TTRB was significantly improved in the experiments, the increased weight is not conducive for practical application. In other words, the idea of simply increasing the modulus of elasticity is undesirable for the application of TTRBs. Since the previously mentioned method has disadvantages such as a cumbersome production process, high cost, and a lack of innovation, there is a clear need to explore a new approach to improve the performance of TTRBs.

The International Table Tennis Federation requires that the natural wood content of the TTRBs should be at least 85%. The National Standardization Administration issued the standard GB/T 23115 [31] to regulate the quality of TTRBs. This standard evaluates TTRBs from three aspects: weight, thickness, and appearance, and it categorizes the TTRBs into two types, namely superior quality and first-class quality. However, this standard is somewhat broad given the wide variety of products on the market. In order to further enhance their application, manufacturers have developed a series of indicators for their respective TTRBs. These include the number of layers, racket face size, weight, thickness, sweet spot area, initial amplitude, vibration attenuation time, handle type, and other conventional evaluations. The most widely used evaluation indicators are categorized according to the player’s style, typically classified into offensive, defensive, and all-round types [32]. Typically, the performance of the TTRB is mainly based on the subjective evaluation of the athletes [33]. So far, well-known brand companies, such as STIGA and Butterfly, characterize TTRBs based on their performance in areas such as controllability, softness, stiffness, flexibility, power, precision, and ease of play (Figure 1a). However, the TTRB species, TTRB and rubber sponge coordination, and the level of the player also affect the table tennis ball track and the player’s feeling [34]. Due to numerous uncertainties, information on the testing methods and analytical studies of TTRBs remain very limited. Lionel et al. [26] conducted a study comparing vibration modes and frequencies obtained through simulations and experimentation, allowing for the validation of a finite element model for the racket blade. Yoichi et al. [35] investigated the effect of racket mass and the stroke rate on the kinematics and kinetics of the trunk and racket arm during a table tennis topspin backhand. Their study found that, regardless of the racket mass, the racket speed at impact was significantly lower at high ball frequencies compared to low frequencies. Peter et al. [28] introduced an approach for estimating ball speed and spin in table tennis using a single racket-mounted inertial sensor. This method is beneficial for both beginners and professional players, aiding in the analysis of techniques and tactics during training and competition. Nicolas et al. [36] investigated the impact of a table tennis ball on the polymeric racket layers under various incident angles and spin conditions. Zhou et al. [37] developed an intelligent table tennis racket with adjustable stiffness based on anisotropic electrorheological elastomers. This smart racket is designed to adapt to different playing styles and the physical conditions of users, standardize the training of unconventional table tennis techniques, and expand the application of electrorheological elastomers (EREs) in smart wearable devices and soft robotics. So far, most research on TTRBs has focused on the biomechanics of table tennis maneuvers and dynamic mechanical analysis using finite element models [38,39,40]. There is still a lack of knowledge regarding the evaluation of TTRBs and the development of effective testing methods.

As a physical vapor deposition (PVD) method [41], magnetron sputtering offers numerous advantages, such as high purity; strong adhesion; high deposition rate; excellent uniformity on large-area substrates; ease of automation; the ability to sputter a wide range of metals, alloys, or compounds; and extensive applicability [42]. Its deposition is achieved by rapidly colliding ionized inert gas atoms with the surface of the negatively biased target under a high electric field, thus inducing the ejection of atoms which then condense on a substrate and eventually generate a membrane [43]. As we know, the structure of biomass materials is typically porous and anisotropic, making it difficult to create a high vacuum environment. Due to advancements in sputtering power supplies and vacuum technology, PVD technology can now be widely applied to biomass substrates [44]. The radio frequency power supply allows the sputtering film to occur in a lower vacuum environment, while molecular pumps can quickly achieve the desired vacuum state. The most commonly used and easily prepared sputtered materials are pure metals, which are produced using single-target magnetron sputtering. This method allows for the tailoring of chemical and physical properties through parameter optimization by using only one target. In recent years, magnetron sputtering metal films applied to the surface of biomass substrates have endowed them with properties such as conductivity, wettability, electromagnetic shielding, etc., expanding their potential application fields. For instance, Jiao et al. [45] reported the development of a core–shell-structured composite consisting of cotton-derived carbon fibers and nano-copper. Li et al. [46] studied the deposition of Cu films with proper thickness on wood surfaces, finding it to be an effective method for achieving a rougher surface texture. Liang et al. [47] fabricated the superamphiphobic-functionalized CuO microflower/Cu(OH)_2_ nanorod array hierarchical structure on a Cu–bamboo surface as a rough coating via a facile alkali-assisted surface oxidation technique. Cai et al. [48] fabricated a self-bonded natural fiber product with high hydrophobic and electromagnetic interference shielding performance, developed using the chemical etching treatment and magnetron sputtering of a Cu film. Wan et al. [49] reported that a Cu film was deposited on the surface of a paper via magnetron sputtering, and the Cu/paper was also electro-oxidized using the cyclic voltammetry method to convert the superficial metallic Cu into the highly electrochemically active Cu_2_O. Inspired by these impactful studies, it is of great importance to explore the use of magnetron sputtering to integrate wood with a metal film for the development of novel TTRBs.

In this paper, we proposed an effective method to deposit Ti layers with varying thicknesses ranging from 0.5 to 5 μm on a seven-ply plywood TTRB panel using magnetron sputtering. In addition, the evaluation criteria for the TTRB are discussed, and the rebound distance detection equipment is designed to evaluate the performance of the TTRB. Dynamic mechanical analysis was used to measure the mechanical properties of samples, while non-contact profilometry and XRD were applied to examine the surface roughness and crystalline structures of the prepared samples. A laser vibrometer was used to analyze and evaluate the vibration behavior and rigidity of the TTRB after being struck by a ping-pong ball.

## 2. Materials and Methods

### 2.1. Materials

The TTRB production process is generally divided into the following steps: material selection, glue plate, drying and aging, and sand milling. Most of the wood is selected with a low output rate of diameter cutting, and the selected plate should be textured straight, arranged densely and evenly, and without color differences, knots, mineral lines, and other defects. The TTRB samples were fabricated using the commonly used materials of Ayous (*Triplochiton scleroxylon*) [50] and Limba (*Terminalia superba*) [51]. The core board and splint board samples of Ayous were prepared with a size of 300 × 200 mm (L × T) and the thickness ranging from 2.5 mm to 1.0 mm. The panel board samples of Limba had a size of 300 × 200 × 0.8 mm (L × T × R). These samples were oven-dried (24 h, 103 ± 2 °C) to a constant weight. All wood materials were purchased from DHS Co., Ltd. (Shanghai, China). Bisphenol epoxy resins with a viscosity of 200–300 mPas were purchased from Dow Co., Ltd. (Midland, Kansas City, MO, USA). The mix ratio of the epoxy and the cure agent was 3:1. The Ti target (99.999% purity) was supplied by Beijing New Material Company Limited. (Beijing, China).

### 2.2. Preparation of Ti Coating on the Wood Surface

All Ti films were deposited with a radio frequency magnetron sputtering system. The target used was a Ti (99.999% purity) plate with a diameter of 5 cm. Sputtering was carried out in argon (Ar-99.995% purity), and the sputtering system was equipped with a diffusion pump backed by a rotary pump to pump down the sputtering system to a base pressure of 3 × 10^−3^ Pa. The thicknesses of the Ti films were checked in situ with a quartz crystal monitor located near the wood substrate during the sputtering process. The wood substrate was pre-conditioned at room temperature, and the sputtering power was 100 W with a fixed target substrate distance of 6 cm.

### 2.3. Preparation of TTRB Samples

The TTRB is processed according to the production procedures of forming, gluing, aging, cold pressing, health preservation, milling, and pasting. Firstly, the Ayous core board is sanded to 2.5 mm thick, and symmetrical cross-cross billets are used, that is, the middle core layer is made of Ayous, the adjacent upper and lower layers are made of Ayous splints, and the upper and lower layers are made of Limba veneers [52]. Secondly, seven-ply plywood samples were made by the DHS Company under the following conditions: 150 g/m^2^ adhesive coverage for each surface; the open assembly time was 15–20 min at room temperature, 1 min/mm cold pressing time at 30 °C, and a pressure of 1.2 MPa [53]. After conditioning at room temperature for 1 day, the plywood was then cut into specimens. The deviation of sample quality and thickness, respectively, does not exceed ±3 g, 0.5 mm, in line with standard GB/T 23115. The results are summarized in Table 1.

### 2.4. Characterization

**Figure 2:**
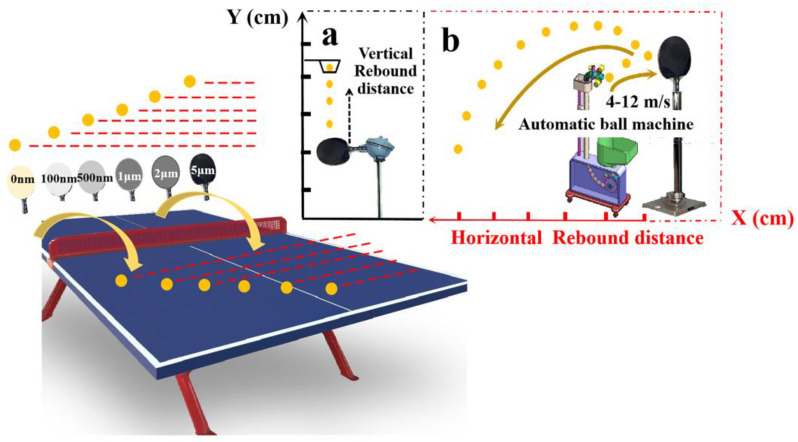
The schematic for the tests of the rebound distance of a ping–pong ball in free fall and the vibration of TTRB (**a**). Dynamic rebound test device (**b**).

The surface structure of the treated samples was determined using X-ray powder diffraction (XRD, Philiphs, PW 1840 diffractometer, Kyoto, Japan), operating with Cu-K radiation at a scan rate of 4°/min, an accelerating voltage of 40 kV, and an applied current of 30 mA ranging from 5° to 80°. Non-contact surface profilometry (Wyko, Greenbackville, VA, USA) was used to calculate the surface roughness [54,55]. Viscoelasticity was measured using the DMA Q800 (TA, Newcastle, DE, USA) analyzer at room temperature. The single cantilever mode was selected, and the measurement was carried out on a rectangular cross-sectional bar with dimensions of 35 × 10 × 0.8 mm^3^ (L × T × R) [56]. Vertical rebound distance (Figure 2a) and dynamic rebound distance (Figure 2b) testing require the TTRB to be pneumatically fixed to the handle. The steps are as follows: Select 4 TTRBs as repeat samples; the weight of each TTRB must not differ by 5 g, and the total weight is within the range of 85~90 g. The weight of each ping-pong ball is controlled at 2.6~2.7 g. The serving speed of the table tennis machine is 4 m/s, 6 m/s, 10 m/s, and 12 m/s, respectively, and the test area is kept closed to reduce the influence of air flow on the test results. Each measurement was performed five times independently of one another.

## 3. Results

### 3.1. XRD Spectrum and Roughness of the Ti Coating on the Wood Surface

**Figure 3:**
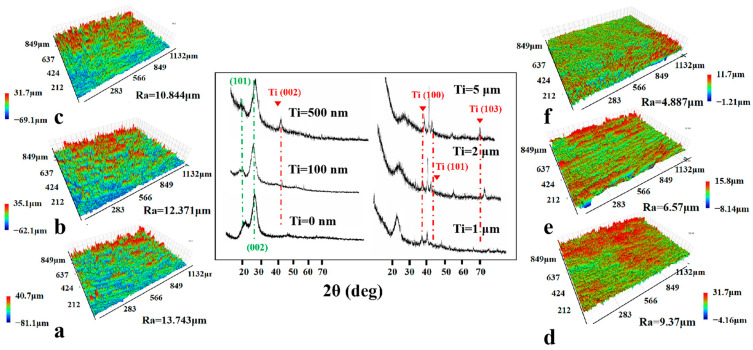
XRD spectra and 3D scan image of the surfaces of the pristine wood (**a**), (**b**–**f**) the Ti−treated wood with different deposition thicknesses of 100 nm, 500 nm, 1 μm, 2 μm, and 5 μm, respectively.

The XRD spectrum of the film is affected by various factors such as sputtering power, temperature, and the substrate material. The effect of film thickness of the Ti film on the phase structure was discussed in Reference. Figure 3 presents the X-ray diffraction patterns of pristine wood and Ti-treated wood samples of varying thicknesses. As a fiber material, the diffraction peaks at 15° and 22° correspond to the (101) and (002) crystal planes of cellulose in the wood [57]. The films of varying thicknesses exhibited a strong Ti (002) orientation. The (100) and (101) diffraction peaks along with the (103) peak at diffraction angles of 34.5°, 40.2°, and 70.9°, respectively, intensified with the increase in film thickness, while other diffractions did not. Thickness affects the degree of crystallization but does not alter the hexagonal structure of the Ti film. Thin film deposition is a continuous and complex process, which includes three stages, i.e., the film-forming process through the island-shaped membrane, mesh membrane, and continuous membrane. The diffusion and migration of sputtered atoms on the surface of the substrate play a crucial role in determining the microstructure of the film.

In order to evaluate the different thicknesses of the film on the surface roughness of the sample, a non-contact surface profiler test method was used. In Figure 3, the three-dimensional image illustrates that the roughness of the original wood surface was 13.74 μm; the blue concave area was more widely distributed, and the concave surface was due to the texture of the wood cell wall and cell cavity [58]. As the thickness of the sputtered film increased, the roughness gradually decreased. The roughness of the 5 μm thick Ti wood was 4.887 μm. There were a few red raised areas on the predominantly green smooth surfaces, indicating that the sputtered nanoparticles were mainly deposited in the cell cavity of the wood, thereby reducing the surface roughness [59]. The changes in roughness revealed that the Ti atoms preferentially deposited on the lower regions of the wood surface. Although the lateral diffusion of energy gradually spread on the surface, the vacancy on the surface was occupied so that the cell cavities of the wood were filled, and the wood surface was flat [60].

### 3.2. Dynamic Mechanical Analysis of a Ti/Wood Composite

**Figure 4:**
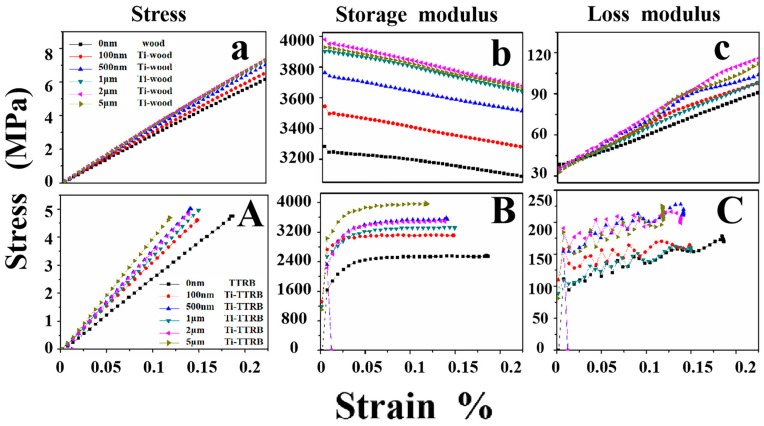
DMA curve of different thicknesses: the Ti–wood (**a**–**c**) and Ti–TTRB (**A**–**C**).

DMA test results, including the stress–strain curves and the storage and loss data, are illustrated in Figure 4 [61]. Figure 4a shows the stress–strain curves of the wood with Ti films of different thicknesses, where the elastic modulus of samples increased significantly due to the increased slope [62]. The elastic modulus of 0 nm, 100 nm, 500 nm, 1 μm, 2 μm, and 5 μm thick film samples were measured as 33 MPa, 36.5 MPa, 34.78 MPa, 40.5 MPa, 41 MPa, and 41.5 MPa, respectively. These changes were due to the dislocations within the grain structure [63]. The interaction of dislocations formed a dislocation network, which inhibited further grain movement and consequently reinforced the films. However, when the Ti film thickness reached 1 μm or more, changes in the Ti–wood elastic modulus became less obvious, and dislocation activity most likely occurred simultaneously with grain boundary slip and diffusion [64]. Furthermore, the dislocations propagated through the grains and were eventually absorbed by the grain boundaries [65]. The storage modulus of the pristine wood was 3200 MPa, increasing by 18% to 3800 MPa for the 5 μm Ti–wood at a strain of 0.015% (Figure 4b). The loss modulus also increased linearly [66]. Thus, the rigidity of the metal film can improve the elastic modulus of wood samples. Based on this result, we aim to make use of these changes in the TTRB. Then, the three-point bending dynamic mechanical tests were performed on the Ti-TTRB samples. In Figure 4a, the elastic moduli of TTRB samples with different thicknesses of Ti films were measured as 22 MPa, 26.5 MPa, 28.2 MPa, 25.5 MPa, 23 MPa, and 22.5 MPa, respectively. It was clearly demonstrated that the stress–strain curve changes, and the Ti-TTRB samples with different thicknesses were irregular. Due to the very high modulus of the TTRB sample, the stiffness of the Ti film had a negligible effect compared to the much larger modulus of the substrate. From another point of view, the storage modulus can be released under alternating strains. However, the storage modulus of TTRB samples in the experiment did not exhibit the consumption state when the strain increased (Figure 4b). This result suggested that the TTRB sample does not yield under the driving force. Therefore, this testing method does not work for TTRBs.

### 3.3. Rebound Distance and TTRB Vibrations

**Figure 5:**
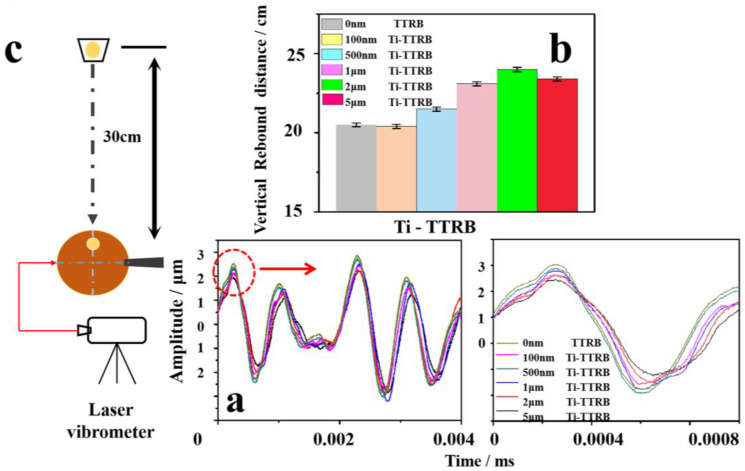
(**a**,**b**) Vibration curve and vertical rebound distance images of TTRBs with different thicknesses of Ti films. (**c**) The schematic diagram of ball and laser vibration measurement.

As shown in Figure 5c, a TTRB testing method was developed, in which the TTRB was fixed on a leveled countertop under a constant force. The table tennis ball was dropped freely from a height of 30 cm to hit the TTRB. The vertical rebound height of the table tennis ball after its first rebound, the vibration image of the TTRB after first hitting, the fall point of the ball, and the vibration point were examined and recorded. As shown in Figure 5a, the TTRB can oscillate around the equilibrium position, reciprocating its vibration after the ball hits it. The vibration attenuation curve was not completely regular, primarily because, when the TTRB was subjected to external forces, the different layers of the TTRB experienced varying stress force superposition effects due to the inhomogeneity of the TTRB. The external force first hits the panel layer, and the hardness of the layer decides how deep to swallow the ball. The term “swallowing” the ball means that the TTRB hits the ball and gives feedback to the athlete’s hand feel [32,67]. The description of the hand feel was also three levels, namely, hard, soft, and control. The hand feel was defined as the initial amplitude of TTRB vibrations, while the amplitude representing the characteristic range and intensity of the vibrations (Figures S1 and S2). In the red circle in Figure 5a, the different thicknesses of the Ti films of the TTRB exhibited varying initial amplitudes, and the amplitude reduced from 2.6 μm (0 nm Ti-TTRB) to 2.1 μm (5 μm Ti-TTRB) as the film thickness increased, indicating that the rigid metal film can effectively improve the hardness of TTRBs (Figure S3). Figure 5b shows the vertical rebound height (VRH) of the ball, showing that as the film thickness increased, the VRH increased from 20.8 cm (pristine TTRB) to 21.9 cm (500 nm Ti film TTRB) and further increased to 23.7 cm (5 μm Ti film TTRB). These results suggest that the compatibility between the Ti film and the TTRB significantly enhances the mechanical performance of the composite.

### 3.4. Dynamic Rebound Test

**Figure 6:**
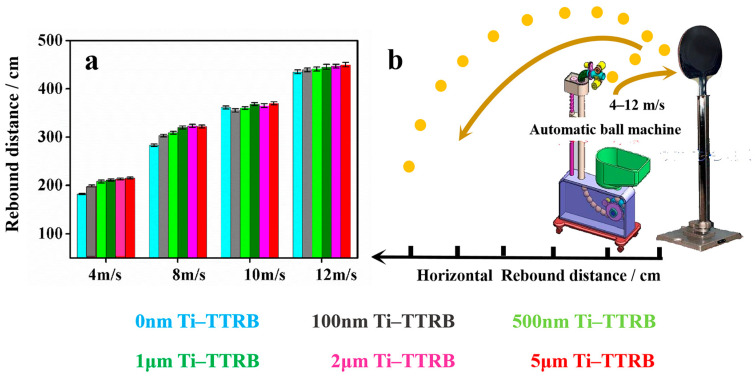
(**a**) Dynamic horizontal rebound distance of the TTRB with different thicknesses of Ti films. (**b**) The schematic diagram of the test rebound distance detection equipment.

This test method simulated the actual use environment of the TTRB. The dynamic rebound device is shown in Figure 6b. The TTRB was hit by the table tennis ball at different speeds from an automatic ball machine. The same quality of table tennis balls was selected for the impact tests. Each test distance datum represents the average rebound distance of 50 balls. The height of the TTRB from the ground was 150 cm. The TTRB was fixed on a vertical countertop under a constant force. The test area remained closed to minimize the impact of air movement on the trajectory of the table tennis ball. Figure 6a shows the horizontal rebound distance (HRD), where the TTRB was hit by the table tennis ball at different speeds. When the ball hits the TTRB at a speed of 4 m/s, the HRDs of TTRBs with different Ti film thicknesses of 0 nm, 100 nm, 500 nm, 1 μm, 2 μm, and 5 μm were 182 cm, 198 cm, 208 cm, 211 cm, 213 cm, and 215 cm, respectively. The rebound distance increased due to the enhanced hardness of the TTRB provided by the metal Ti film. Meanwhile, when the ball speed was 8 m/s, the Ti-TTRB displayed the same advantage in rebound distance compared to the pristine TTRB. The rebound rates of the TTRB with five different Ti film thicknesses increased by 7%, 9%, 13%, 14%, and 13.7%, respectively. However, when the ball speeds were 10 m/s and 12 m/s, the effect of Ti film hardness on the improvement in the rebound distance of the TTRB was negligible. The main reason probably was due to the special structure of the TTRB. When the TTRB suffered a greater impact, the impact from the external delivery concentrated in the middle of the core board, causing all the energy of the TTRB to be activated (Figure S4). Although the metal film had a certain hardness, in a high-intensity application environment, the surface hardness could not enhance the overall hardness of the TTRB.

## 4. Conclusions

In this study, an easy and efficient method for TTRB surface hardening was developed through the magnetron sputtering process. The effects of the thickness of the Ti film on the wood surface were explored. Compared to the pristine wood, the modulus of the wood with different Ti coating thicknesses increased by 5.1% to 24%. Furthermore, three types of TTRB mechanical property testing devices were developed, including free fall rebound, laser vibration, and dynamic rebound tests. The results indicate that the VRH of the different thicknesses of Ti-TTRB increased by 5% to 13%, and the amplitude reduced by 12% to 19% due to the rigidity of the Ti film. The 5 μm thick Ti films deposited on the TTRB surface exhibited the best mechanical properties, with the initial amplitude and VRH reaching 2.1 μm and 23.7 cm, respectively. Moreover, in the dynamic rebound test, the rebound rate increased by 13.7% when the ball speed was 8 m/s. However, as the hitting speed increased, the rigidity effect of the Ti film gradually decreased. When the ball speed was above 10 m/s, there was almost no difference in the rebound rate of TTRB with different thicknesses of Ti films. In conclusion, the panel board is a key factor for determining the hardness of the TTRB, which is mainly reflected by two indicators: initial amplitude and rebound distance. The increased hardness of the TTRB helps improve the speed of the ball and the player’s hitting feeling, which is suitable for offensive players. The findings in this work pave the way for innovative developments in TTRBs and the unification of TTRB evaluation standards.

## Figures and Tables

**Table 1:** List of samples characterized.

Sample	0 nm-Ti TTRB	100 nm-Ti TTRB	500 nm-Ti TTRB	1 μm-Ti TTRB	2 μm-Ti TTRB	5 μm-Ti TTRB
Weight (g)	85 g ± 1	84 g ± 2	84 g ± 2	85 g ± 2	86 g ± 3	89 g ± 2
Thickness (mm)	6.3 ± 0.2	6.4 ± 0.1	6.3 ± 0.3	6.5 ± 0.3	6.3 ± 0.2	6.4 ± 0.1

## Data Availability

The original contributions presented in this study are included in the article and Supplementary Material. Further inquiries can be directed to the corresponding author.

## Supplementary Materials

The following supporting information can be downloaded at https://www.mdpi.com/article/10.3390/polym17050583/s1, Table S1: List of samples characterized; Figure S1: Vertical rebound distance images of TTRB of different brand types; Figure S2: Vibration curve images of TTRB of different brand types; Figure S3: Vibration curve images of TTRB of different brand types; Figure S4: Dynamic horizontal rebound distance of TTRB with different brand types.
