# Supplementary material for: An Investigation of the Mechanical Properties of Ti Films Reinforced with Wood Composites by Growing Ti Particles on a Wood Substrate
Source: Polymers (Basel). 2025 Feb 22;17(5):583. doi: 10.3390/polym17050583 (PMC11902493; doi:10.3390/polym17050583)
Supplement: Supplementary file 1 [file polymers-17-00583-s001.zip › polymers-3474489-supplementary.pdf]

polymers

Supplementary Information

An Investigation of the Mechanical Properties of Ti Films Reinforced with Wood Composites by Growing Ti Particles on a Wood Substrate

Wenhui Bao [1,2,3], Yini Tan [1], Ziyi Ying [1], Rui Xue [1], Xiaojiang Xu [1], Shuangping Duan [1,2], Haizhuan Lin [1,2] and Hui Chen [1,2,*]

[1] College of Architecture and Energy Engineering, Wenzhou University of Technology, Wenzhou 325035, China; nefuwenhui@163.com (W.B.); tanyini007@163.com (Y.T.); 20210421@wzut.edu.cn (Z.Y.); wwxrpig@163.com (R.X.); 20210420@wzut.edu.cn (X.X.); shp_duan@126.com (S.D.); linhaizhuan@wzut.edu.cn (H.L.)
[2] Wenzhou Key Laboratory of Intelligent Lifeline Protection and Emergency Technology for Resilient City, Wenzhou 325035, China
[3] Key Laboratory of Bio-based Material Science & Technology, Northeast Forestry University, Ministry of Education, Hexing Road 26, Harbin 150040, China
* Correspondence: chenhui0306@wzu.edu.cn

Table S1. List of samples characterized.

Sample	STIGA-CLCR	STIGA-Rosewood	DHS-DIUT	DONIC-2000	5μm-Ti TTRB
Weight (g)	92g±5	91g±5	91g±3	85g±2	89g±2
Thickness (mm)	6.8±0.2	6.9±0.1	6.1±0.1	6.5±0.3	6.4±0.1

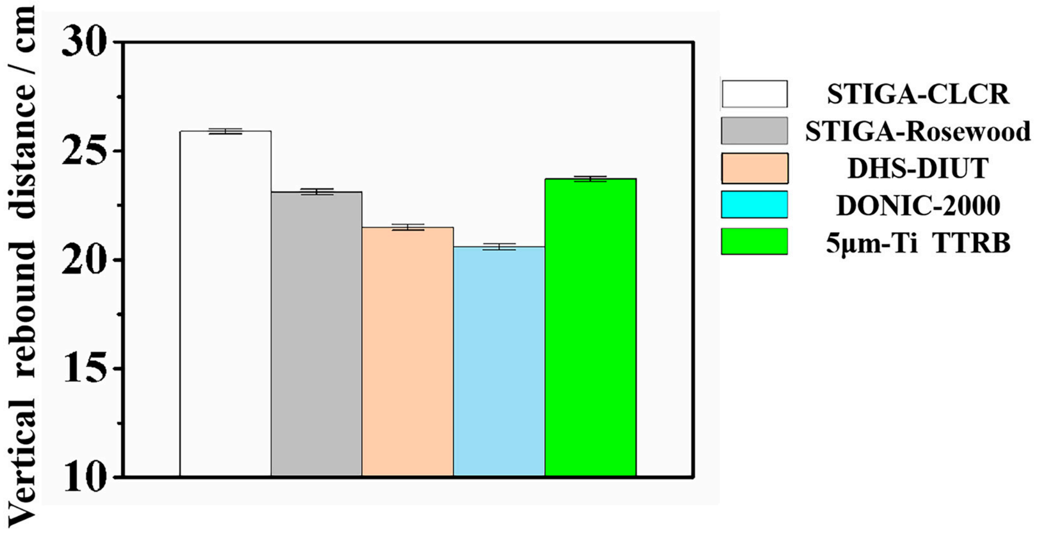

Figure S1. Vertical rebound distance images of TTRB of different brand types.

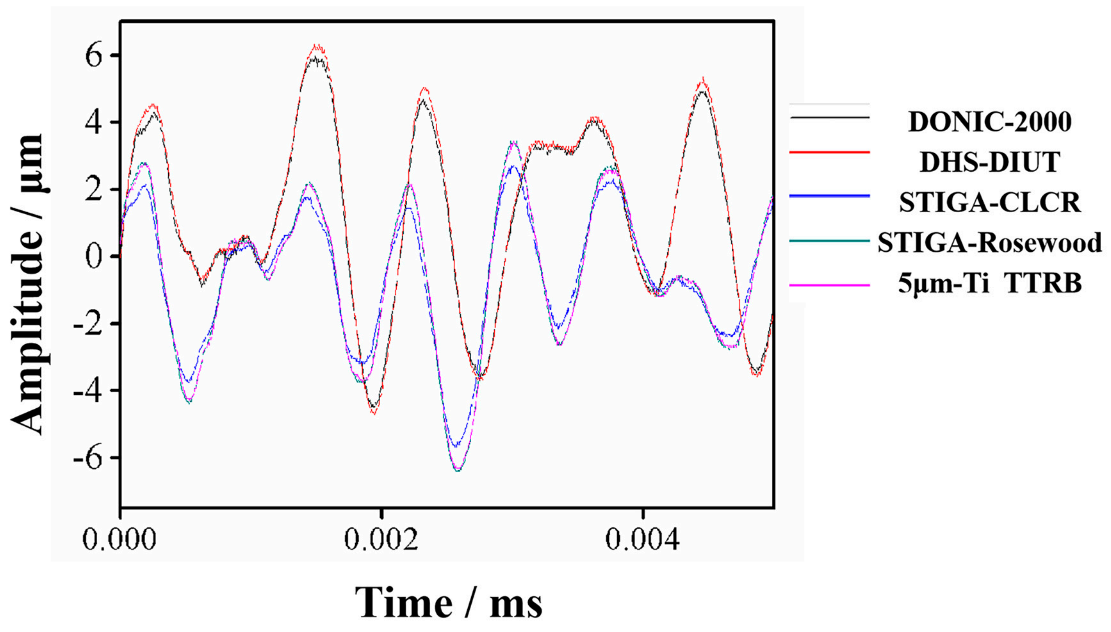

Figure S2. Vibration curve images of TTRB of different brand types.

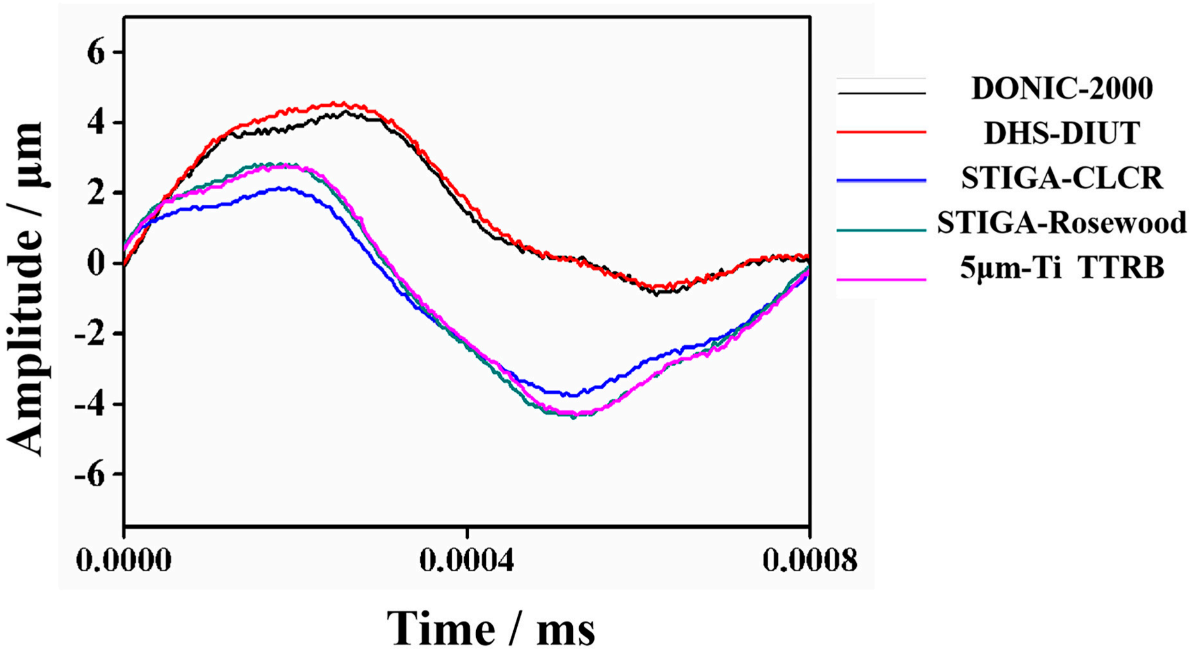

Figure S3. Vibration curve images of TTRB of different brand types.

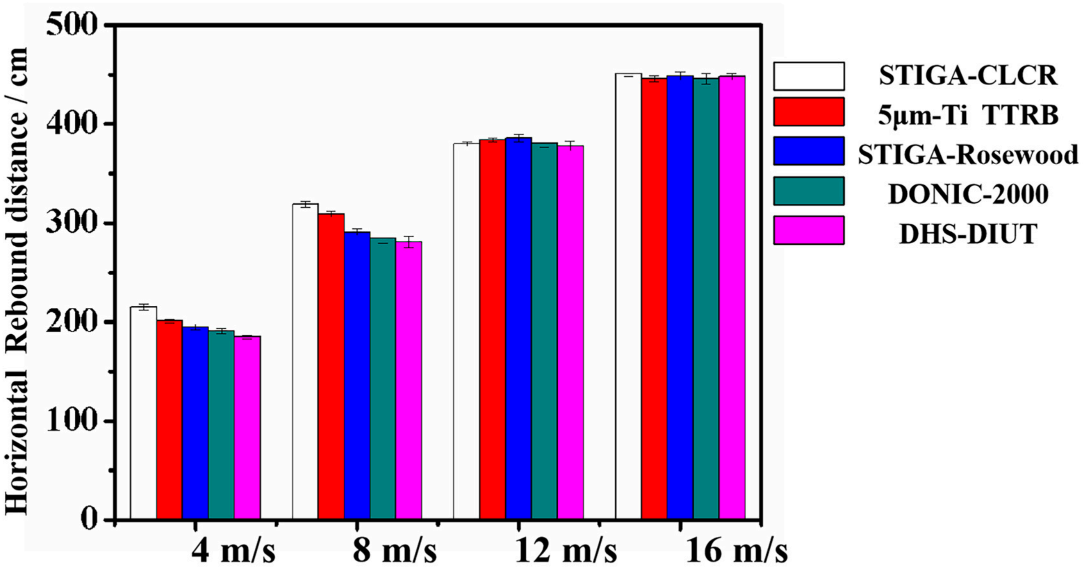

Figure S4. Dynamic horizontal rebound distance of TTRB with different brand types.
